# Real-Time Underwater Wireless Optical Communication System Based on LEDs and Estimation of Maximum Communication Distance

**DOI:** 10.3390/s23177649

**Published:** 2023-09-04

**Authors:** Minglun Zhang, Hongyu Zhou

**Affiliations:** 1 State Key Laboratory of Information Photonics and Optical Communications, Beijing University of Posts and Telecommunications, Beijing 100876, China; zhouhongyu@bupt.edu.cn; 2 School of Electronic Engineering, Beijing University of Posts and Telecommunications, Beijing 100876, China

**Keywords:** optical propagation, optical transmitters, optical receivers, underwater wireless optical communication, visible light communication

## Abstract

This paper presents a real-time underwater wireless optical communication (UWOC) system. The transmitter of our UWOC system is equipped with four blue LEDs, and we have implemented pre-emphasis technology to extend the modulation bandwidth of these LEDs. At the receiver end, a 3 mm diameter APD is utilized. Both the transmitter and receiver are housed in watertight chassis and are submerged in a water pool to conduct real-time underwater experiments. Through these experiments, we have obtained impressive results. The data rate achieved by our system reaches up to 135 Mbps, with a BER of 5.9 × 10^−3^, at a distance of 10 m. Additionally, we have developed a convenient method for measuring the underwater attenuation coefficient, using which we have found the attenuation coefficient of the water in experiments to be 0.289 dB/m. Furthermore, we propose a technique to estimate the maximum communication distance of an on–off keying UWOC system with intersymbol interference, based on the *Q* factor. By applying this method, we conclude that under the same water quality conditions, our system can achieve a maximum communication distance of 25.4 m at 80 Mbps. Overall, our research showcases the successful implementation of a real-time UWOC system, along with novel methods for measuring the underwater attenuation coefficient and estimating the maximum communication distance.

## 1. Introduction

In recent years, underwater wireless optical communication (UWOC) has gained significant research interest due to its higher speed and moderate distance capabilities compared to acoustic communication and radio frequency (RF) technologies [1,2]. UWOC can be categorized into two types based on the light sources used: laser diode (LD) based and light-emitting diode (LED) based. LD-based UWOC offers a higher data rate and smaller beam divergence angle, resulting in an extended communication distance. However, the smaller beam divergence angle makes alignment more challenging. On the other hand, LED-based UWOC has a lower data rate and larger beam divergence angle, which leads to higher channel attenuation and limited communication distance. However, it is easier to align. In many scenarios, a data rate of tens of Mbps is sufficient to meet application requirements, making LED-based UWOC suitable [1].

Over the years, various advancements have been made in UWOC technology. For instance, in 2010, AquaOptical II was developed, enabling underwater communications over 50 m at a low signal-to-noise ratio [3]. In 2013, the adoption of discrete multitone (DMT) modulation technology allowed error-free underwater communication of 58 Mbps [4]. In 2018, a field programmable gate array (FPGA)-based underwater communication system was implemented, enabling real-time communication at a distance of 10 m with a data rate of 25 Mbps [5]. The same year witnessed the utilization of the photomultiplier tube (PMT) in UWOC systems [6]. In 2019, offline high-speed underwater communication experiments exceeding Gbps were successfully completed [7]. In 2020, an FPGA-based UWOC system capable of full-duplex real-time communication was developed [8]. Additionally, the silicon photomultiplier (SiPM) found its application in UWOC systems in the same year [9]. In 2021, a prototype for underwater video transmission based on UWOC was realized [10]. Furthermore, in 2022, a UWOC system based on FPGA and quadrature amplitude modulation (QAM)-orthogonal frequency-division multiplexing (OFDM) technology was developed [11]. Finally, in 2023, a three-stage cascaded T-bridge equalizer was designed to expand the 3 dB bandwidth of the LED [12]. More details regarding these LED-based UWOC systems can be found in Table 1. We can notice UWOC systems can be categorized into real-time and offline systems. Contrasting with real-time systems, offline UWOC systems rely on MATLAB for signal modulation, arbitrary wave generators for waveform output, oscilloscopes for sampling, and MATLAB for demodulation. Presently, real-time UWOC systems have inferior signal processing capabilities compared to offline systems, leading to a noticeable discrepancy in the data rate between the two types. Real-time systems are closer to practical deployment, while offline systems present an intriguing future for UWOC.

With regards to the modulation format, offline systems often employ complex modulation formats to achieve higher communication data rates [13,14,15]. On the other hand, real-time systems prefer simpler modulation formats such as on–off keying (OOK) and frequency-shift keying (FSK) [16,17]. The OOK signal has a relatively wide spectrum, allowing power to be maintained above the −3 dB bandwidth. Thus, the amplitude-frequency responses of the channel within this range affect the system’s data rate. Although serious intersymbol interference (ISI) may occur in this system, a high data rate, usually over three times the value of the −3 dB bandwidth, can be obtained as long as the noise is sufficiently small [18]. It is important to note that data rate is measured in bps and bandwidth is measured in Hz.

This paper presents a real-time LED-based UWOC system. The transmitter consists of four LEDs, and pre-emphasis technology is utilized to extend the LED’s bandwidth to 40.3 MHz. At the receiver end, a 3 mm diameter large area avalanche photodiode (APD) is employed to obtain a large field of view (FOV) angle. The transmitter and receiver are enclosed in watertight chassis and immersed in a 10 m water pool to conduct real-time UWOC experiments, achieving a maximum data rate of 135 Mbps.

Furthermore, this paper introduces a convenient method for measuring the attenuation coefficient and proposes a method to infer the maximum communication distance based on eye height and receiver noise. In UWOC research, a practical challenge lies in obtaining a sufficiently long-distance water pool for testing. Additionally, due to the utilization of LEDs as the light source with a large beam angle, the use of reflective mirrors to redirect a collimated beam, as commonly performed in LDs UWOC, is not feasible. Therefore, it becomes crucial to theoretically infer the maximum possible communication distance. Our paper presents a method to estimate this distance, providing valuable insights for UWOC researchers. By considering the eye height at 80 Mbps and receiver noise, it is possible to estimate that the maximum communication distance at this data rate is 25.4 m.

The subsequent sections of this paper are organized as follows: the second section describes the design of the transmitter and receiver, along with their performance verification in the air. The third section elaborates on the underwater experiments and presents a measurement method for the water attenuation coefficient. In the fourth section, the underwater experiments are discussed and a method for calculating the maximum communication distance based on the *Q*-factor is proposed. Lastly, the fifth section concludes the paper.

## 2. Module Design and Verification

### 2.1. Transmitter Design

**Figure 1:**
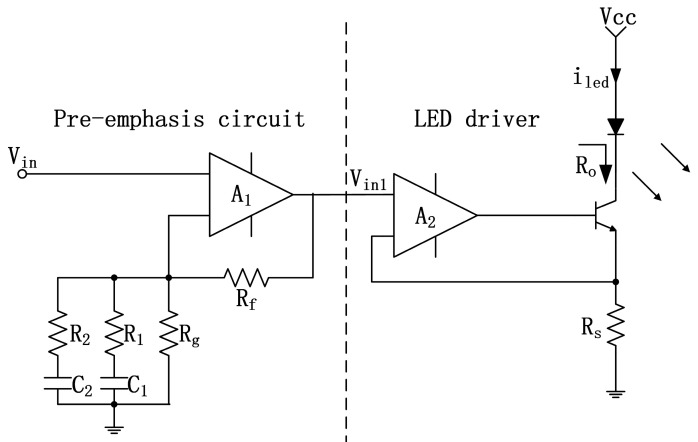
The proposed LED driver with a 2nd order pre-emphasis circuit (Ref. [18], Figure 1 and Ref. [19], Figure 10).

In this experiment, we aim to achieve a data rate in the range of tens of Mbps while maximizing the communication distance. To optimize the light intensity, we have chosen high-power blue LEDs and incorporated spot LED lenses. Specifically, we are using the GD CS8PM1.14 LEDs from OSRAM. These LEDs have a peak wavelength of 451 nm and a half-power angle of 80°. At a forward current of 350 mA, they emit an optical power of 641 mW, which increases to 2.5 times the optical power at the maximum forward current of 1 A. However, due to the large junction area, the modulation bandwidth of the GD CS8PM1.14 is limited to approximately 6.94 MHz. To overcome this limitation and extend the bandwidth, we have implemented a 2nd order pre-emphasis circuit [18,19]. The pre-emphasis and drive circuit can be seen in Figure 1. In this circuit, R_1_, R_2_, C_1_, and C_2_ are used to pre-emphasize the driving signal by applying different gains to different frequency components of the signal.

### 2.2. Receiver Design

The alignment of a transmitter and receiver submerged in water is more complex compared to the alignment in air, as it lacks the support of an underwater tripod. To address this, we have increased the field of view angle of the receiver. This was achieved by incorporating the Hamamatsu S8664-30K photodetector, which features a large photosensitive area with a 3 mm diameter. Consequently, this results in a larger junction capacitance of 22 pF and a nominal bandwidth of 140 MHz. In the amplifier circuit, we utilize the LTC6268-10 from Analog Devices.

**Figure 2:**
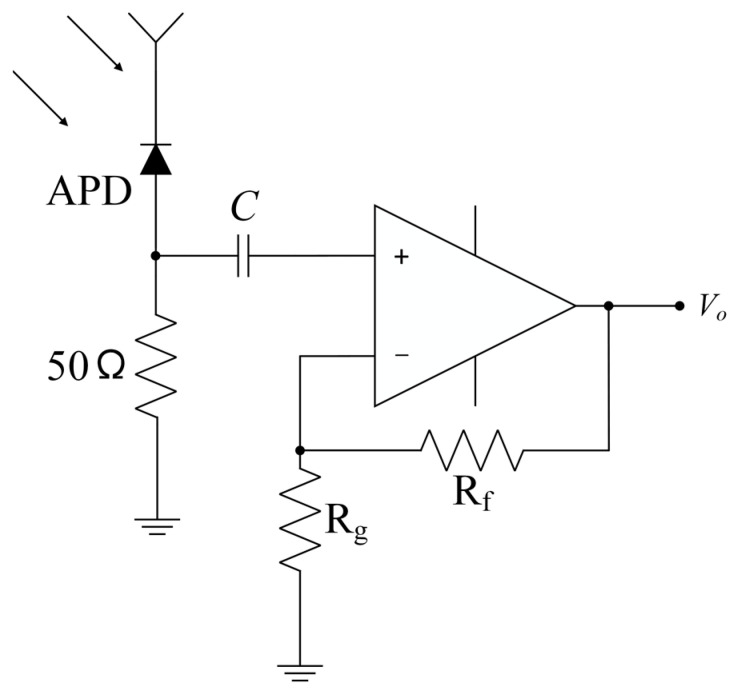
Receiver topology.

The non-inverting amplifier topology, demonstrated in Figure 2, is adopted for its input impedance that surpasses ten times the resistance of 50 Ω. As a result, no load effect is formed, allowing this topology to be considered as a cascade of two-stage systems. We can calculate the −3 dB bandwidth for both circuits separately, and subsequently determine the overall bandwidth using Equation (1) [20], where $f_{1}$ is the bandwidth of System 1, $f_{2}$ is the bandwidth of System 2, and $f_{-3\;dB}$ is the bandwidth of the cascaded system.

(1) $$\frac{1}{f_{-3\mathrm{dB}}}=1.1\sqrt{\frac{1}{f_{1}^{2}}+\frac{1}{f_{2}^{2}}}$$

APD junction capacitance and 50 Ω resistance form a low-pass filter. Its bandwidth can be obtained from (2), where $R_{s}=50\;\Omega$, $C_{apd}=22\;pF$. So, the bandwidth is 145 MHz.

(2) $$f_{-3dB}=\frac{1}{2\pi R_{s}C_{apd}}$$

Considering that the gain–bandwidth product (GBP) of LTC6268-10 is 4 GHz [21], and the gain of the non-inverting amplifier is set to 90, we can deduce that the bandwidth of the non-inverting amplifier is 44.4 MHz. By referring to Equation (1), the overall bandwidth of the receiver is determined to be 38.5 MHz.

### 2.3. Performance Verification of Transceiver Module

After completing the pre-emphasis circuit, we conducted several tests to evaluate the performance of both the transmitter and the receiver. These tests included examining the frequency response, analyzing the eye diagram, measuring the receiver’s output voltage noise in a dark environment, and assessing the system’s bit error rate (BER).

**Figure 3:**
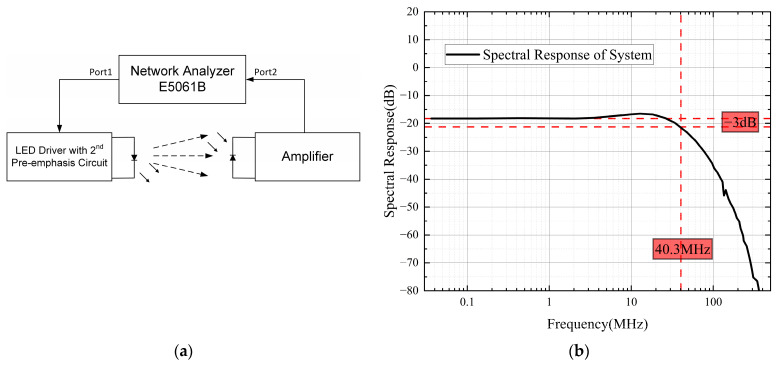
Frequency response measurement and result. (**a**) Block diagram for frequency response measurement. (**b**) Frequency response of our system.

To assess the frequency response, we employed a test block diagram as shown in Figure 3a. The bandwidth of the system was determined to be 40.3 MHz, as depicted in Figure 3b.

**Figure 4:**
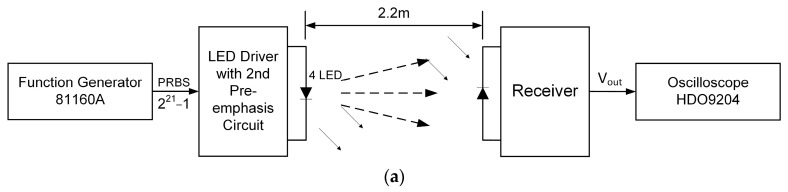

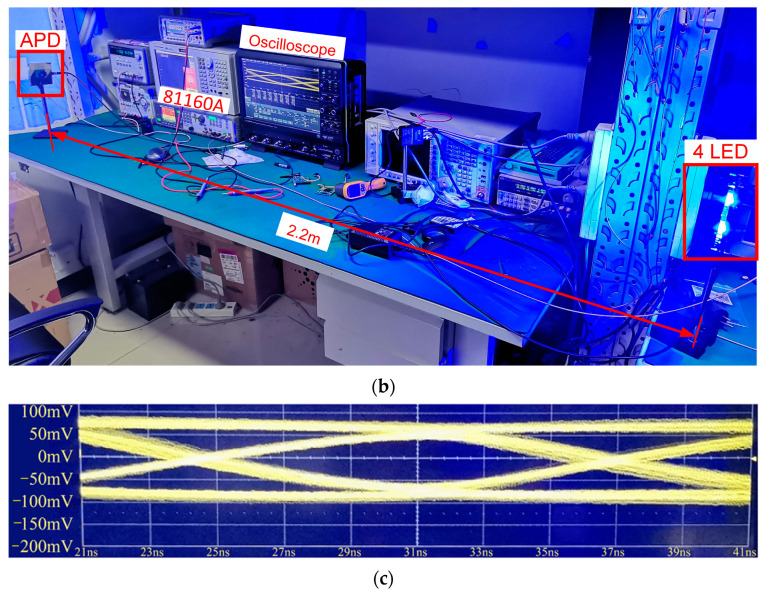
Eye diagram measurement. (**a**) Block diagram for eye diagram measurement. (**b**) Experiment setup for eye diagram measurement. (**c**) Eye diagram of our system.

For the measurement of the eye diagram, we utilized the block diagram illustrated in Figure 4a. The experimental field setup, presented in Figure 4b, involved placing the transmitter and receiver 2.2 m apart without using any lens or optical filter. The resulting eye diagram is shown in Figure 4c.

**Figure 5:**
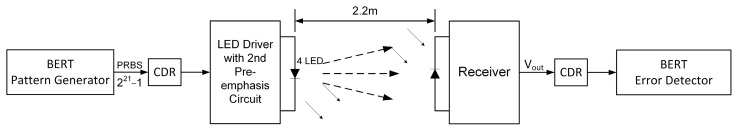
Block diagram for the BER test.

To conduct the BER test, as depicted in Figure 5, we positioned the transmitter and receiver 2.2 m apart while omitting the use of a lens or optical filter. Indoor lighting was turned on during this test. Our bit error rate tester (BERT) lacked a signal amplitude adjustment function. To adjust the amplitude of the pseudo-random binary sequence (PRBS) signal, we utilized a self-made clock and data recovery module (CDR). In the receiver, another CDR was employed to recover the data and clock from the received signal. Consequently, we achieved a BER of 0 at a data rate of 80 Mbps over a duration of ten minutes.

To measure the receiver’s output voltage noise, we followed the procedure outlined below: we turned off the indoor lighting, sealed the receiver with a c-mount cover, and placed it in a black bag, leaving only the power line and output signal line connected to an oscilloscope. The standard deviation of the receiver’s output voltage noise was determined to be 1.335 mV.

### 2.4. Lenses for LEDs and APD

**Figure 6:**
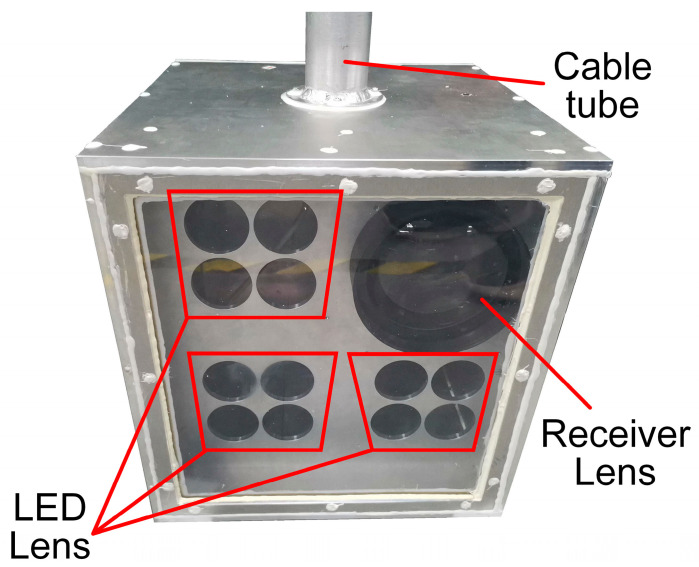
Chassis for the transmitter and receiver.

The transmitter boards employ the F12985 LED lens from LEDiL, featuring a nominal beam angle of 4.9°. Figure 6 illustrates the arrangement of the four LEDs on each transmitter board. On the other hand, the receiver is equipped with a lens that has a diameter of 120 mm and a focal length of 160 mm.

In each chassis, three transmitter boards and one receiver board are incorporated as shown in the diagram. The chassis also features a hollow aluminum tube on top, which facilitates the passage of the power line and signal line for external power supply and testing. To ensure waterproofing, all screw holes and gaps on the chassis are sealed with waterproof glue. Additionally, an optical window made of transparent acrylic plate is positioned at the front of the chassis.

**Figure 7:**
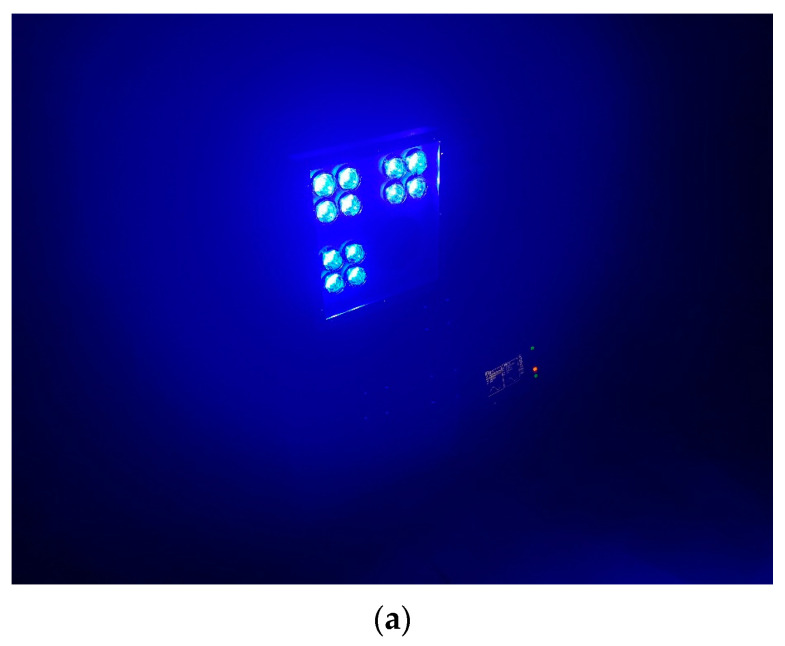

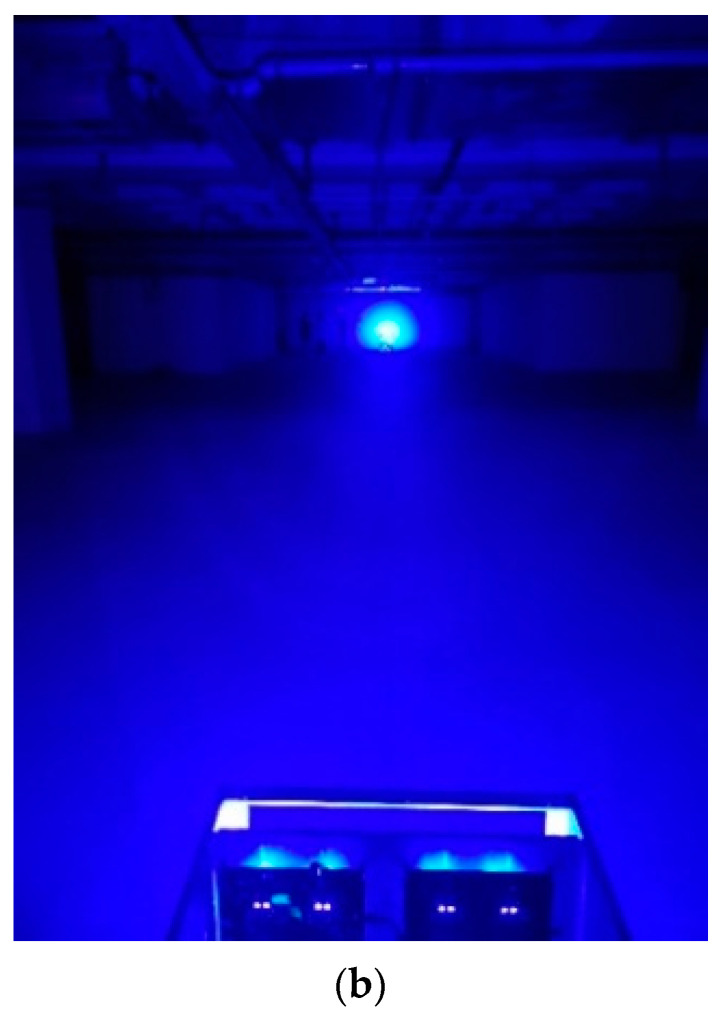
Beam angle measurement. (**a**) Light source. (**b**) Light spot.

Figure 7 displays the measurements of the beam angle conducted in a 30 m garage. In this test, all 12 LEDs were illuminated, resulting in a light spot with a diameter of approximately 2.6 m, as depicted in Figure 7b. Consequently, we determined the beam angle to be approximately 4.96°, which closely aligns with the lens’s nominal value. This larger angle proves advantageous for achieving accurate alignment between the transmitter and receiver.

### 2.5. Transmitter and Receiver Design Summary

The device models and specific parameters of the transmitter and receiver are shown in Table 2.

## 3. Underwater Experimental Setup and Results

### 3.1. Measurement of Underwater Attenuation Coefficient

The experiment took place in an outdoor environment, using a black inflatable polyvinyl chloride (PVC) pool filled with water for the lawn. Due to the duration of the experiment spanning several days, dust and leaves fell into the water, resulting in lower optical transmittance compared to clear water. Two chassis were utilized in the experiment, one acting as the transmitter and the other as the receiver.

**Figure 8:**
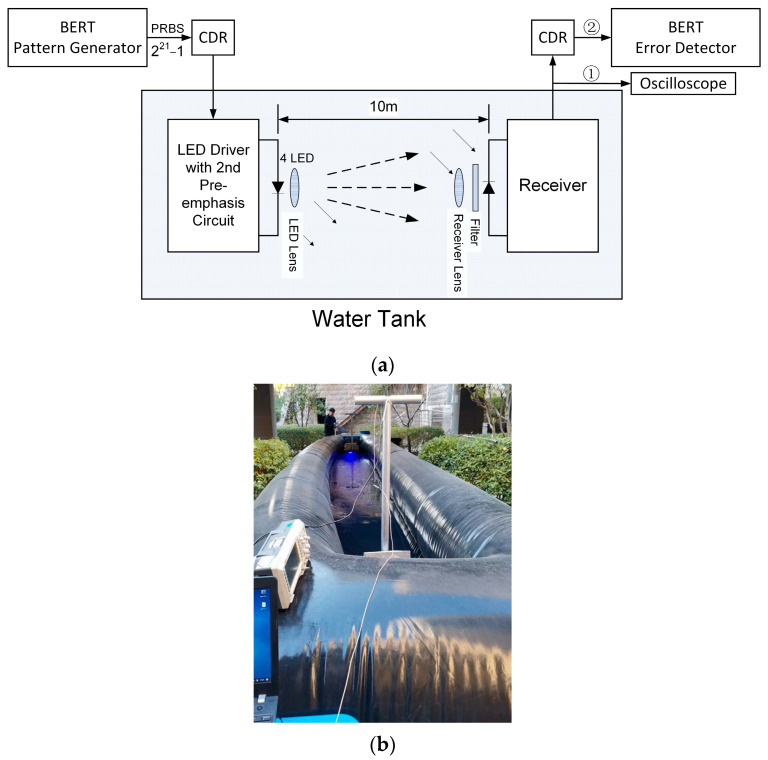
Underwater experiments (**a**) Block diagram for underwater experiments (**b**) Field experiments setup.

Figure 8 illustrates the block diagram of the experiment. A BERT sends a PRBS21 signal, which is then adjusted in amplitude through a self-made CDR and fed into a transmitter board. In this specific experiment, we employed four LEDs to provide sufficient illumination for the received signal at a rate of 80 Mbps. The LED bias current was set to 1 A, and the alternating current (AC) current was 0.67 A. The output signal from the receiver is connected to an oscilloscope in order to observe the eye diagram. Additionally, the signal is also connected to another CDR and the BERT’s error detector to measure the BER.

The system in Figure 6 integrates transmitter and receiver. In our experiment, we employed two of these systems to facilitate one-way communication. It is worth noting that the transmitter and receiver in the opposite direction are identical. During the one-way communication experiment, only the transmitter is working in one system, and only the receiver is working in the other system.

To determine the attenuation coefficient of the water, we initially positioned the two chassis on the ground beside the pool, maintaining a distance of 10 m between them, which precisely matched the length between the two chassis within the pool. Subsequently, we measured the eye height of the receiver’s output signal. It is important to note that angle adjustment can be a challenging task. The pitch angle adjustment involves placing multiple layers of small sheets underneath the front or rear of the chassis, while the horizontal adjustment is achieved by rotating the chassis. In order to determine the optimal pitch angle and horizontal direction, we used an oscilloscope to observe the eye diagram of the receiver. We considered the adjustment to be successful when the eye diagram displayed the maximum opening, indicating that the optimal pitch angle or horizontal angle had been achieved. The observed maximum eye height was 600 mV. Next, we immersed the two chassis in the pool with a separation of 10 m, while ensuring that the water level was approximately 10 cm above the top of the chassis. Again, we employed the same alignment method used previously to align the transmitter and receiver. The maximum eye height underwater was measured at 308 mV.

The light emitted from the LEDs towards the receiver undergoes three types of attenuation: geometric attenuation, dielectric attenuation, and optical attenuation. Geometric attenuation is caused by the divergence of the light beam, while dielectric attenuation results from absorption, scattering, and other factors related to the air or water. Optical attenuation occurs due to the presence of optical lenses and windows along the optical path. According to [22], in a wireless optical communication system, the received power is

(3) $$P_{r}=\frac{m+1}{2\pi }\frac{A\cos\psi }{d^{2}}\cos^{m}\phi T_{s}(\psi )g(\psi )P_{t}$$

where $P_{r}$ is the received optical power by the photodetector, m is the mode number of the light source, A is the physical detector area, d is the distance between the light source and the photodetector, ϕ is the transmitter’s emergence angle, ψ is the receiver’s incidence angle, $T_{s}\left(\psi \right)$ is the signal transmission of the filter, $g\left(\psi \right)$ is the concentrator gain, and $P_{t}$ is the transmitted optical power of the light source. Essentially, (3) describes both geometric and optical attenuation. Assuming m, A, d, ϕ=ψ=0, $T_{s}\left(0\right)$, and $g\left(0\right)$ are given constants, $P_{r}$ is directly proportional to $P_{t}$. Simplifying further, (4) is obtained.

(4) $$P_{ra}=K_{g}\times K_{o}\times exp(-c_{a}d)\times P_{t}$$

where $K_{g}$ and $K_{o}$ are scale factors for geometric attenuation and optical attenuation. $P_{ra}$ is the received power in the air, $c_{a}$ is the atmosphere attenuation coefficient [1,16]. In the air, the eye height observed at the receiver is

(5) $$H_{a}=ℜMR\left(P_{ra1}-P_{ra0}\right)=K_{g}\times K_{o}\times exp(-c_{a}d)\times ℜMR\left(P_{t1}-P_{t0}\right)$$

where $H_{a}$ is the eye height in the air, R is the responsivity of the photodetector, M is the avalanche gain of APD, R is the transimpedance gain of the transimpedance amplifier, $P_{ra1}$ is the received optical power for ‘1′ s in the air, $P_{ra0}$ is the received optical power for ‘0′ s in the air, $P_{t1}$ is the transmitted optical power for ‘1′ s, $P_{t0}$ is the transmitted optical power for ‘0′ s. Since the attenuation in 10 m of air is negligible, $exp\left(-c_{a}d\right)$ can be approximated as 1. Then

(6) $$H_{a}\approx K_{g}\times K_{o}\times ℜMR\left(P_{t1}-P_{t0}\right)$$

Similarly, the eye height in water is

(7) $$H_{w}=K_{g}\times K_{o}\times exp\left(-c_{w}d\right)\times ℜMR\left(P_{t1}-P_{t0}\right)$$

where $H_{w}$ is eye height in water, $c_{w}$ is attenuation coefficient in water. When the transmitter and receiver are precisely aligned, $K_{g}$ and $K_{o}$ are nearly identical to those in the air.

By dividing both sides of (6) and (7) separately, (8) is obtained.

(8) $$\frac{H_{a}}{H_{w}}=exp\left(c_{w}d\right)$$

So

(9) $$c_{w}=\frac{1}{d}\ln\frac{H_{a}}{H_{w}}$$

Substitute d=10 m, $H_{a}=600\;mV$, and $H_{w}=308\;mV$ into (9), we find that $c_{w}=0.0667/m$. Alternatively, $c_{w,dB}=0.289\;dB/m$. It is worth noting that selecting a lower data rate can mitigate the influence of ISI when measuring the eye height. However, such measures are unnecessary in this case.

The attenuation coefficient of pure water ranges from approximately 0.04/m to 0.05/m. However, the attenuation coefficient of tap water varies significantly depending on the impurity content, which is much more than 0.05/m, that is 0.217 dB/m [1,2,23]. Taking into account the water source used in our experiment, as well as the fact that it was exposed to outdoor conditions for several days, the measured attenuation coefficient falls within the expected range for tap water. Therefore, we can confidently deem the measurement results as reliable.

### 3.2. Underwater Wireless Optical Communication Experiments and Results

We conducted an experiment in an outdoor pool to measure the BER and eye height for underwater communication at different rates. The results are summarized in Table 3. In this field, the commonly used BER standard is 3.8 × 10^−3^, as the error correction encoding algorithm can correct it to 1 × 10^−9^, which is a widely adopted BER standard in optical communication. However, achieving precise control of the BER at exactly 3.8 × 10^−3^ in our experiment proved to be quite challenging. As a result, we opted for the nearest BER of 5.9 × 10^−3^.

It is important to note that our system has a limited bandwidth of 40.3 MHz. As the data rate increases, we observed a more significant impact from ISI and noticeable jitter. This effect is independent of the water attenuation. Consequently, ISI prevents us from achieving higher data rates.

Since the experiment took place during the daytime, we encountered interference from ambient light, which affected the receiver. As a result, the standard deviation of the receiver output noise increased to 3.2 mV. Background light plays a crucial role in UWOC, and its impact on UWOC systems is influenced by various factors. These factors include the illumination of background light, the field of view angle of the receiver (which depends on the size of the photodetector photosensitive surface and the focal length of the focusing lens), as well as the optical aperture of the receiver’s lens, among others. In order to comprehensively assess the background light situation, we measured the output noise of the receiver without emitting any optical signals. This measurement reflects the combined influence of the aforementioned factors. To isolate the influence of the noise of the optical receiver itself, we also conducted measurements in a dark environment, as shown in Table 2.

## 4. Estimation of Maximum Communication Distance

Due to the size of the pool, we cannot experiment with channel lengths longer than 10 m. However, we can estimate the maximum communication distance theoretically. In order to evaluate the communication distance at a data rate of 80 Mbps, which is related to the BER, we will use the *Q*-factor. Simultaneously, the *Q*-factor decreases as the communication distance increases.

The relationship between the *Q*-factor and the BER is [24]

(10) $$P_{e}(D_{opt})=\frac{1}{2}erfc(\frac{Q}{\sqrt{2}})$$

where $P_{e}\left(D_{opt}\right)$ is the minimum BER at the optimum decision level $D_{opt}$, and Q is defined as

(11) $$Q=\frac{\mu_{1}-\mu_{0}}{\sigma_{1}+\sigma_{0}}$$

where $\mu_{1}$ ($\mu_{0}$) is the mean of “1” s (“0” s) and $\sigma_{1}$ ($\sigma_{0}$) is the standard deviation of “1” s (“0” s).

**Figure 9:**
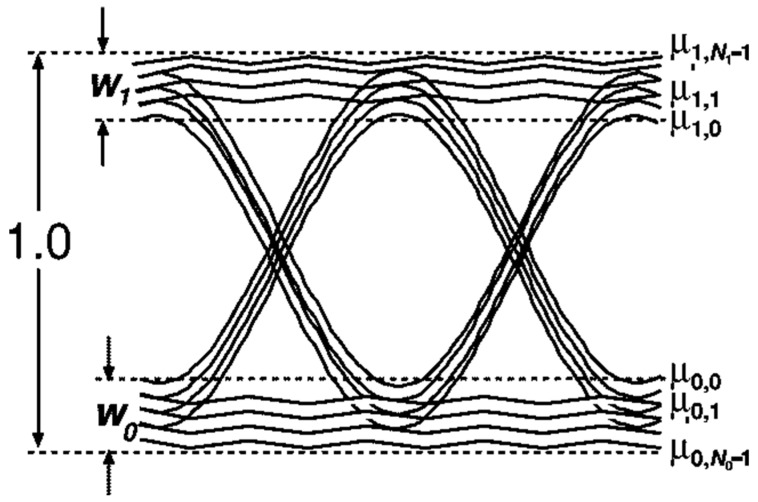
Noiseless eye diagram in the presence of ISI (Ref. [24], Figure 1).

Since the bandwidth in our system is only 40.3 MHz and the data rate is 80 Mbps, there is a serious issue of ISI. Therefore, we refer to the method outlined in [24] to evaluate the maximum communication distance. As shown in Figure 9, due to ISI, “0” s (“1” s) split into several rails. Additive Gaussian white noise superimposes on each “0” s and “1” s rail, respectively. The distance from the bottom rail to the top rail is normalized to 1.0. The mean values of these rails are defined as $\mu_{0,0}$, $\mu_{0,1}$,…, $\mu_{0,N_{0}-1}$ for “0” s, and $\mu_{1,0}$, $\mu_{1,1}$,…, $\mu_{1,N_{1}-1}$ for “1” s. The standard deviations for rails are defined as $\sigma_{0,0}$, $\sigma_{0,1}$,…, $\sigma_{0,N_{0}-1}$ for “0” s, and $\sigma_{1,0}$, $\sigma_{1,1}$,…, $\sigma_{1,N_{1}-1}$ for “1” s, respectively. The probabilities of occurrence for each ‘0’ rail and ‘1’ rail are set to be $p_{0,j}$ and $p_{1,j}$, respectively, which satisfies

(12) $$\sum_{j=0}^{N_{0}-1}p_{0,j}=\sum_{j=0}^{N_{1}-1}p_{1,j}=1$$

According to [24,25,26,27], the BER is

(13) $$P_{e}(D)=\sum_{j=0}^{N_{0}-1}P_{0,j}(D)+\sum_{j=0}^{N_{1}-1}P_{1,j}(D)$$

where $P_{e}\left(D\right)$ is the BER for decision level D, $p_{0,j}\left(D\right)$ and $p_{1,j}\left(D\right)$ are defined as

(14) $$P_{0,j}(D)=\frac{p_{0,j}}{4}erfc(\frac{D-\mu_{0,j}}{\sqrt{2}\sigma_{0,j}})$$

(15) $$P_{1,j}(D)=\frac{p_{1,j}}{4}erfc(\frac{\mu_{1,j}-D}{\sqrt{2}\sigma_{1,j}})$$

$erfc\left(x\right)$ is decreasing on $\left(-\infty ,+\infty \right)$, so

(16) $$P_{e}(D)<\sum_{j=0}^{N_{0}-1}\frac{p_{0,j}}{4}erfc(\frac{D-\mu_{0,0}}{\sqrt{2}\sigma_{0,j}})+\sum_{j=0}^{N_{1}-1}\frac{p_{1,j}}{4}erfc(\frac{\mu_{1,0}-D}{\sqrt{2}\sigma_{1,j}})={P^{\prime }}_{e}(D)$$

Assume $\sigma_{0,j}=\sigma_{1,j}=\sigma$, so

(17) $${P^{\prime }}_{e}(D)=erfc(\frac{D-\mu_{0,0}}{\sqrt{2}\sigma })\sum_{j=0}^{N_{0}-1}\frac{p_{0,j}}{4}+erfc(\frac{\mu_{1,0}-D}{\sqrt{2}\sigma })\sum_{j=0}^{N_{1}-1}\frac{p_{1,j}}{4}$$

Substitute (12) into (17)

(18) $${P^{\prime }}_{e}(D)=\frac{1}{4}erfc(\frac{D-\mu_{0,0}}{\sqrt{2}\sigma })+\frac{1}{4}erfc(\frac{\mu_{1,0}-D}{\sqrt{2}\sigma })$$

Assume $D-\mu_{0,0}=\mu_{1,0}-D$, then

(19) $${P^{\prime }}_{e}(D)=\frac{1}{2}erfc(\frac{D-\mu_{0,0}}{\sqrt{2}\sigma })$$

Define $Q^{\prime }$ as

(20) $$Q^{\prime }=\frac{D-\mu_{0,0}}{\sigma }=\frac{\mu_{1,0}-D}{\sigma }=\frac{\mu_{1,0}-\mu_{0,0}}{2\sigma }$$

So

(21) $$P_{e}(D)<\frac{1}{2}erfc(\frac{Q^{\prime }}{\sqrt{2}})={P^{\prime }}_{e}(D)$$

At the receiver, $Q^{\prime }$ observed on an oscilloscope is

(22) $$Q^{\prime }=\frac{\mu_{1,0}-\mu_{0,0}}{2\sigma }=\frac{ℜMR(P_{r1,0}-P_{r0,0})}{2\sigma }$$

Taking water attenuation into consideration, (3) can be rewritten as

(23) $$P_{r}=P_{t}\frac{m+1}{2\pi }\frac{A}{d^{2}}\cos^{m}\phi \cos\psi T_{s}(\psi )g(\psi )exp\left(-c_{w}d\right)$$

When ϕ=ψ=0, and m, A, $T_{s}\left(0\right)$, $g\left(0\right)$ is given, $P_{r}$ is proportional to $P_{t}$ as

(24) $$P_{r}=kP_{t}\frac{exp\left(-c_{w}d\right)}{d^{2}}$$

where

(25) $$k=\frac{m+1}{2\pi }AT_{s}(0)g(0)$$

By substituting (24) into (22), we obtain

(26) $$Q^{\prime }=\frac{ℜMRk(P_{t1,0}-P_{t0,0})}{2\sigma }\frac{exp\left(-c_{w}d\right)}{d^{2}}$$

Assuming $P_{e}^{\prime }\left(D\right)=3.8\times 10^{-3}$, according to (21), $Q^{\prime }=2.67$. We denote this specific $Q^{\prime }$ value as $Q_{min}^{\prime }$. $Q_{min}^{\prime }$ corresponds to the maximum communication distance $d_{max}$. When conducting an UWOC experiment with a channel length of $d_{1}$, we obtain a *Q*-factor denoted as $Q_{1}^{\prime }$. Then

(27) $$\frac{{Q^{\prime }}_{1}}{{Q^{\prime }}_{min}}=\frac{\frac{ℜMRk(P_{t1,0}-P_{t0,0})}{2\sigma }\frac{exp\left(-c_{w}d_{1}\right)}{d_{1}{}^{2}}}{\frac{ℜMRk(P_{t1,0}-P_{t0,0})}{2\sigma }\frac{exp\left(-c_{w}d_{max}\right)}{d_{max}{}^{2}}}=exp\left[c_{w}\left(d_{max}-d_{1}\right)\right]\frac{d_{max}{}^{2}}{d_{1}{}^{2}}$$

Equation (27) can be rewritten as

(28) $$\frac{{Q^{\prime }}_{1}}{{Q^{\prime }}_{min}}=exp\left[c_{w}\left(d_{max}-d_{1}\right)\right]\frac{d_{max}{}^{2}}{d_{1}{}^{2}}$$

In our experiments, $c_{w}=0.0667/m$, $d_{1}=10\;m$, Q′=3083.2×2=48.125, and $Q_{min}^{\prime }=2.67$. By substituting these values into (28) and solving the equation, we find that $d_{max}=25.4\;m$. Consequently, we can conclude that, under a bit error rate standard of 3.8 × 10^−3^, the maximum communication distance for water with an attenuation coefficient of 0.0667/m at a data rate of 80 Mbps is 25.4 m. Many researchers do not have a sufficiently long water pool, so estimating the maximum communication distance from an experimental result with a limited transmission distance is a common problem. These researchers can benefit from Formula (28), which is not found in the previous literature.

## 5. Conclusions

In this paper, we have designed an LED-based real-time UWOC system. We conducted BER measurements at a distance of 10 m for various communication rates. Among them, at a communication rate of 135 Mbps, the BER was found to be 5.9 × 10^−3^. To facilitate further research, we have provided detailed parameters of the optical transmitter and receiver.

Additionally, we propose a method in this paper for measuring the attenuation coefficient of water. This method proves to be convenient for researchers exploring unknown bodies of water. Through our experiments, we determined that the attenuation coefficient of water used was 0.289 dB/m.

Furthermore, considering the limited availability of experimental conditions for long distance underwater communication, we present a method to estimate the maximum communication distance for UWOC systems. This method takes into account the effects of ISI and is suitable for UWOC systems based on OOK modulation. By using this method, we find that under the same experimental water quality conditions, the UWOC system described in this paper can achieve a maximum communication distance of 25.4 m at a communication rate of 80 Mbps.

## Figures and Tables

**Table 1:** Comparison of the UWOC systems based on LED.

Distance	Data Rate	BER	Power	Source	Wavelength	Detector	Channel Type	Note	Year/Ref
50 m	2.28 Mbps		10 W_O_	Blue LED	470 nm	APD	Olympic size pool	Real time	2010/[3]
2.5 m	58 Mbps	0	10.5 W_E_	Blue LED	470 nm	APD	very-pure water	Offline	2013/[4]
10 m	25 Mbps	1.0 × 10^−4^		Blue LED	448 nm	APD	tap water	Real time	2018/[5]
10 m	2 Mbps	3.8 × 10^−3^		Blue LED	440 nm	PMT	simulated harbor water	Offline	2018/[6]
1.2 m	3 Gbps	3.8 × 10^−3^	120 mW_O_	Blue LED	446.4 nm	PIN	tap water	Offline	2019/[7]
10 m	1 Mbps	2.9 × 10^−3^	10.07 mW_O_	Blue LED	445 nm	APD	tap water	Real time	2020/[8]
28 m	20 Mbps	10^−4^	600 mW_O_	Blue LED	470 nm	SiPM	clear water	Real time	2020/[9]
10 m	50 Mbps								
46 m	2.5 Mbps		3 W_O_	Blue LED	458 nm	APD	air	Real time	2021/[10]
5 m							outdoor diving pool		
5 m	50 Mbps	3.359 × 10^−3^	3 W_E_ × 7	Green LED		APD		Real time	2022/[11]
1 m	50 Mbps	8.0 × 10^−5^		Green LED		APD	tap water	Real time	2023/[12]
10 m	80 Mbps	0	1.9 W_O_	Blue LED	451 nm	APD	water for the lawn	Real time	This work
	100 Mbps	0							
	120 Mbps	1.0 × 10^−7^							
	135 Mbps	5.9 × 10^−3^							
	140 Mbps	8.0 × 10^−3^							

Where the W_E_ is the unit of electrical power, the W_O_ is the unit of optical power.

**Table 2:** Transmitter and receiver design summary.

**Transmitter**	LED	GD CS8PM1.14
	Peak wavelength	451 nm
	LED lens	F12985
	Beam angle (measured)	4.96°
**Receiver**	APD	S8664-30K
	OP AMP	LTC6268-10
	Transimpedance gain	4500 Ω
	Receiver aperture	120 mm
	Standard deviation of output voltage noise (measured in a dark environment)	1.335 mV
	Standard deviation of output voltage noise (measured in the work environment)	3.2 mV
**System**	Overall bandwidth (measured)	40.3 MHz

**Table 3:** Experiment results.

Data Rate	BER	Eye Height
80 Mbps	0	308 mV
100 Mbps	0	190 mV
120 Mbps	1.0 × 10^−7^	68 mV
135 Mbps	5.9 × 10^−3^	52 mV
140 Mbps	8.0 × 10^−3^	30 mV

## Data Availability

The data presented in this study are available on request from the corresponding author.
